# Integrated Management of *Aphis craccivora* in Cowpea Using Intercropping and Entomopathogenic Fungi under Field Conditions

**DOI:** 10.3390/jof6020060

**Published:** 2020-05-11

**Authors:** Allan Mweke, Komivi Senyo Akutse, Christian Ulrichs, Komi Kouma Mokpokpo Fiaboe, Nguya Kalemba Maniania, Sunday Ekesi

**Affiliations:** 1 International Centre of Insect Physiology and Ecology (icipe), P.O. Box 30772-00100 Nairobi, Kenya; muekea@gmail.com (A.M.); nguyaman45@yahoo.com (N.K.M.); sekesi@icipe.org (S.E.); 2 Division Urban Plant Ecophysiology, Faculty of Life Sciences, Humboldt-Universität zu Berlin, Lentzeallee 55–57, 14195 Berlin, Germany; christian.ulrichs@hu-berlin.de; 3 Department of Animal Health and Production, School of Pure and Applied Sciences, Mount Kenya University, P.O. Box 342-01000 Thika, Kenya; 4 International Institute of Tropical Agriculture (IITA), P.O. Box 2008 (Messa) Yaoundé, Cameroon; K.Fiaboe@cgiar.org

**Keywords:** cowpea aphid, *Metarhizium anisopliae*, yield, Duduthrin, biopesticide, damage

## Abstract

Cowpea aphid, *Aphis craccivora*, is a major cowpea pest. Cowpea–cereal intercrop alone does not effectively manage the pest. Use of pesticides in intercrop leads to health and environmental risks. Fungal-based biopesticides offer a better option because they are environment- and consumer-friendly. This study assessed the combined effect of *Metarhizium anisopliae* ICIPE 62 and cowpea–maize intercrop against *A. craccivora* under six treatments: (1) untreated cowpea monocrop, (2) untreated cowpea–maize intercrop, (3) cowpea monocrop + ICIPE 62, (4) cowpea–maize intercrop + ICIPE 62, (5) cowpea monocrop + Duduthrin insecticide, and (6) cowpea–maize intercrop + Duduthrin during three seasons (long rainy/cold and dry/short rainy). In the cold and dry season, cowpea–maize intercrop treated with ICIPE 62 recorded the lowest infestation/cowpea damage, whereas the leaf yield was comparable to cowpea monocrop treated with ICIPE 62. In the short rainy season, the cowpea–maize intercrop treated with ICIPE 62 recorded the lowest infestation/damage, whereas leaf yield was similar to cowpea–maize intercrop treated with ICIPE 62 in the cold and dry season. Duduthrin in monocrop and intercrop did not reduce aphid infestation/cowpea damage levels in all the seasons. Although the efficacy of *M. anisopliae* ICIPE 62-based biopesticide could be affected by seasons, it successfully controlled aphid population in cowpea–maize intercrop under field conditions without affecting aphid-associated natural enemies.

## 1. Introduction

Cowpea (*Vigna unguiculata* L. Walp) is primarily a tropical crop that originated in Africa and has spread to other parts of the world [1]. The crop is mostly grown as an intercrop with cereals, and farmers can harvest it even when cereals failed due to inadequate rainfall because it is drought-tolerant [2,3,4]. The crop is an important leafy vegetable and a valuable source of affordable proteins, vitamins, and income to rural households [5,6]. In Kenya, cowpea is one of the most important indigenous vegetable in terms of production and consumption [7,8]. The area under production of these indigenous vegetables has been increasing [9].

Cowpea aphid, *Aphis craccivora* Koch is a polyphagous pest of cowpea that attacks the crop, feeding on all plant parts and leading to significant yield losses [10,11,12]. Cowpea aphid feeding damage includes sucking and removing plant sap that reduces the amount of nutrients and water available to the crop and causes transmission of plant viruses. Aphid feeding induces symptoms that include chlorosis and stunting that delays onset of flowering and even plant death when infestations are high especially at seedling stage [10,12]. Among the management strategies of *A. craccivora*, the use of chemical insecticides is ranked first by farmers because there are many chemical insecticides registered for use in management of aphids [13]. Synthetic pyrethroids including cypermethrin, alphacypermethrin, deltamethrin, and lambdacyhalothrin are the most commonly used chemical pesticides against sucking insects. Lambdacyhalothrin-based insecticides are used in management of aphids in Kenya and come under different trade names such as Duduthrin (Twiga Chemical Industries Ltd., Nairobi, Kenya), and are easy to access and use. Although aphids are susceptible to pesticides, application of the chemicals does not always result in effective suppression of their population because of their high fecundity, and also they have been reported to develop resistance to some of these chemicals [14,15,16,17]. Moreover, synthetic chemicals pose health risks due to toxic residues, especially on leafy vegetables that are harvested regularly [11,17,18], killing natural enemies of *A. craccivora* and leading to pest resurgence and the need for further pesticide application [14,19]. Intercropping cowpea with cereals such as maize, sorghum, and millet has been used as a strategy in the management of cowpea insect pests including *A. craccivora*, although intercropping alone does not completely control the pest [4,20,21]. Therefore, enhancement of this strategy by monitored application of insecticides has been shown to offer benefits to farmers, especially where cowpea is grown as a grain legume [22,23]. However, where cowpea is grown as a leafy vegetable application of synthetic insecticides increases the risk of consuming pesticide residues because the leaves are harvested regularly and hence there is need to adopt safer pest management approaches.

Different groups of entomopathogenic fungi (EPF) are known to cause high pathogenicity to different insect pests, and their use as biological control agents can reduce reliance on chemical insecticides [24,25,26,27,28]. Use of EPF-based biopesticides in management of *A. cracivora* in vegetables is a good and sustainable alternative to synthetic insecticides because the pest is susceptible to various species of entomopathogenic fungi, and also due to food safety concerns associated with synthetic pesticides, especially where cowpea is grown as a leafy vegetable [29,30]. Several species of *Metarhizium* have been identified as being pathogenic to *A. craccivora*, both in laboratory and in field conditions [30,31]. Furthermore, use of EPF-based biopesticides, notably *Metarhizium anisopliae* ICIPE 62, has been recently demonstrated to suppress aphid populations in cowpea under field conditions [17]. Several EPF-based biopesticide products for management of some aphids species are available in Europe and America, but are less common in Africa, and none have been registered for use against *A. craccivora* [17,32].

Biopesticides derived from EPF have the advantages of being compatible with integrated pest management (IPM) strategies [33], although their low efficacy under field conditions slows down their widespread use. Performance of EPF-based biopesticides can therefore be enhanced by using them in combination with other pest control strategies such as cultural control. Although EPF-based biopesticides have been recommended for use in IPM, no previous studies have been carried out to assess their efficacy when combined with intercropping cowpea and maize in control of *A. craccivora* [25,34]. This study therefore evaluated the efficacy of combining *M. anisopliae* ICIPE 62 application and cowpea–maize intercrop in management of *A. craccivora* under field conditions.

## 2. Materials and Methods

### 2.1. Fungal Culture and Inoculum Preparation

The fungus *Metarhizium anisopliae* isolate ICIPE 62 with a known pathogenicity to *A. craccivora* [17] was obtained from *icipe’s* Arthropod Pathology Unit Germplasm Centre and used in this study.

To mass produce the fungus prior to experiments, long grain rice in Milner bags (60 cm × 35 cm) were sterilized by autoclaving them for 1 h at 120 °C and used as substrate for mass production of fungal conidia. The autoclaved substrate was cooled to room temperature in plastic buckets 35 (Ø) × 25 (width) × 15 cm (depth) before inoculating with a 3-day-old culture of blastopores (50 mL), after which it was covered with sterile polyethylene bags. The inoculated substrate culture was incubated for 21 days at ambient conditions (20–26 °C, 40–70% relative humidity (RH)) [35]. After the incubation period, the bags were removed then dried at room temperature for 5 days. Conidia were harvested by sifting the substrate through a sieve (295 μm mesh size) and stored in a refrigerator (4–6 °C, 40–50% RH) before being used in field experiments. The fungus viability was evaluated before field treatment application by spread-plating 100 µL of conidial suspension at a concentration of 3 × 10^6^ conidia·mL^−1^ in Sabouraud dextrose agar (SDA) plates. The plates were then incubated at 26 ± 2 °C in darkness for 18 h, after which percent fungal spore germination was determined by counting randomly 100 selected conidia on a cover slip under a light microscope (400×) [34]. The conidia germ tubes that were at least as long as twice the diameter of the conidium were scored as viable or germinated [36]. Conidial germination was >90% after 18 h on SDA and was considered adequate for use in the field trials. Conidia concentration per gram was determined by dissolving 0.1 g of conidia in 10 mL of sterile Triton water (0.05% Triton X-100), then serial diluted to 100×, after which the mixture was vortexed for 5 min at about 700 rpm to break conidial clumps and ensure a homogenous suspension. After vortexing, 1 mL of the suspension was pipetted into a hemocytometer, and spore counts were conducted under a light microscope using a Neubauer hemacytometer. The amount of conidia in grams required to produce a concentration of 1 × 10^12^ conidia·ml^−1^ was determined from spores in 0.1 mL.

### 2.2. Lambdacyhalothrin Duduthrin 1.75EC (Twiga Chemical Indutries Ltd.)

The pesticide used in this study was one commonly used in Kenya to control aphids, known as Duduthrin, with lambdacyhalothrin as active ingredient, and was acquired from local agro-chemical outlets in Nairobi, Kenya. Before treatment application, 65 mL of the pesticide was mixed with 20 L of clean water in a knapsack sprayer, 0.05% (1 mL) Integra (sticker, Greenlife Crop Protection Africa Ltd.) was added, and the mixture was thoroughly mixed before application.

### 2.3. Experimental Sites

The experiment was carried out at *icipe’s* Mbita point campus, Homabay County, Western Kenya, located at 00.42931S, 034.20604 E, 1140 m above sea level (m.a.s.l) for three seasons. There are four cropping seasons in Kenya: hot and dry season (January–February), long rainy season (March–June), cold and dry season (July–August), and short rainy season (October–December) [37]. During the long rainy season, the experiment was carried out between March and June 2016. The mean annual rainfall in the long rainy season was ≈130 mm, while the minimum and maximum temperatures were 20 °C and 25.2 °C, respectively, with relative humidity ranging between 60% and 70%. In the cold and dry season, the mean rainfall was ≈60 mm, while minimum and maximum temperatures were 23.7 °C and 29.5 °C, respectively, and relative humidity ranged between 60% and 65%. In the short rainy season, the mean rainfall was ≈80 mm, and the minimum and maximum temperatures were 25.8 °C and 29.3 °C, respectively, while relative humidity ranged between 65% and 70% [38].

### 2.4. Soil Type in the Experimental Site

All the experiments were laid out in one expansive field with uniform soil type, and during every season, the experiment was carried out in a different portion of the same field following the same crop rotation practices. The soil type in the field, as described by Rachilo and Wataka [39], is non-saline, non-sodic, moderately well drained, dark, deep to very grey firm stratified strongly calcareous, cracking, sandy clay to clay loam. On the basis of the soil mapping in the field station carried out by Rachilo and Wataka [39], the authors confirmed that there was no variability in soil type and characteristics at the selected study area.

### 2.5. Crop

The land was prepared by ploughing and harrowing before planting. Aphid-susceptible cowpea landrace (Ex-Luanda) obtained from *icipe’s* germplasm collection was used in the experiment. Maize variety PHB 3253 (Pioneer Hi-Bred Kenya Limited) was used in this study and was acquired from local agro-vet shops. Cowpea was planted in alternate rows with maize in plots measuring 10 m × 10 m; spacing for cowpea was 20 cm intra-row by 75 cm inter-row, with two seeds sown per hole and later thinned to one plant at 14 days after emergence. Spacing for maize was 30 cm intra-row and 90 cm inter rows. Each plot had six experimental units/treatments, making a total of 600 m^2^ per block. Overhead irrigation (sprinkler) was used for the first 3 weeks to support the crops, as it was relatively dry in January during planting, and irrigation discontinued after the onset of the rains. Weeding was done twice a month before the crop established and smothered the weeds, and the frequency of weeding was reduced to once a month. The crop was left for natural aphid infestation.

### 2.6. Treatments, Layout and Design

The experiments were carried out for three consecutive seasons and for each season, one experimental field was planted. The experimental plot had six treatments as follows: (i) untreated cowpea monocrop (UCM), (ii) untreated cowpea–maize intercrop (UCMI), (iii) cowpea monocrop treated with *M. anisopliae* ICIPE 62 (CM62), (iv) cowpea–maize intercrop treated with *M. anisopliae* ICIPE 62 (CMI62), (v) cowpea monocrop treated with Duduthrin (CMD), and (vi) cowpea–maize intercrop treated with Duduthrin (CMID). Emulsifiable formulation of the fungus was used at the concentration of 1 × 10^12^ conidia·mL^−1^ and the spores were suspended in in vegetable oil-elianto (Elianto, Bidco Africa Ltd.) containing 0.05% Integra (sticker, Greenlife Crop Protection Africa Ltd.) with 0.1% nutrient agar, 0.1% glycerin, and 0.5% molasses added as protectants and attractant, respectively [35] The insecticide Duduthrin l.75 EC was applied at the rate of 1.75 g (Active Ingredient (AI)) ha^−1^ with 0.1% Integra. Control treatment were sprayed with water containing 0.05% Integra, 0.1% nutrient agar, 0.05% molasses, and 0.1% glycerin without any fungal conidia and any insecticide solution. In the long rainy season, the spray applications started on day 56 after planting due to late aphid infestation, and thereafter was done on a weekly basis for a period of 6 weeks. For the dry and cold and short rainy seasons, treatment application began 21 days after planting because aphid infestation occurred early compared to the wet season. Treatment applications were also done weekly for 6 weeks. The fungus formulations and the insecticide were applied with different knapsack sprayers with target output of 350 L·ha^−1^, and spraying was done late in the evening between 17:00 and 18:00 h. The experimental design was a randomized block design with four replications per treatment.

### 2.7. Evaluation of Treatments

#### 2.7.1. Aphid Infestation Assessment

Two leaflets, each from the base and the top from 20 randomly selected cowpea plants in the middle rows, were sampled from each plot for aphid infestation assessment. The aphids were dislodged from each host plant with a fine hairbrush into a vial containing 70% ethanol, labelled, and thereafter counted in the laboratory. Aphid colonies were assessed prior to collecting the aphids for counting at the laboratory. Sampling was done on weekly basis from day 7 after planting until the cowpea leaves began to dry out. Aphid infestation on cowpea plants in different treatments were assessed weekly using the following scale: 1–5 rating scale (1 = a few individual aphids, 2 = few small individual colonies, 3 = several small colonies, 4 = large individual colonies, 5 = large continuous colonies) [40].

#### 2.7.2. Assessment of Aphid Mortality Induced by EPF

Mortality of aphids induced by the fungus was assessed weekly before treatment application by picking 30 aphids from each fungal-treated plot and transferring them into plastic dishes (11.3 cm (Ø) × 4 cm (depth)) lined with moist filter paper and placing sterilized cowpea leaves in the dishes to serve as food for the sampled aphids. Whole cowpea leaves were sterilized with 1% sodium hypochlorite and rinsed three times in distilled water for approximately 3 min and allowed to dry in a sterile laminar flow chamber before supplying them to the aphids. Muslin cloth with apertures (300 µm × 300 µm) was placed around the container mouth before placing the cover to allow free air circulation. The dishes were kept at room temperature and mortality was observed daily for 1 week. The leaves serving as food for the aphid were removed and replaced with fresh ones daily. Dead aphids were collected and placed in Petri dishes with sterilized moist filter papers and kept at room temperature before observation under the dissecting microscope for mycosis assessment.

#### 2.7.3. Leaf Damage Assessment

Leaves were sampled weekly from 20 randomly selected cowpea plants from the middle rows in each treatment plot, and sampling and assessment were performed as described above. Leaf damage (infestation and leaf quality and fitness for human consumption) assessment was performed visually using the following scaled developed by Benchasri [41]: 0 = visual damage on leaves and flower buds <10%, 1 = visual damage on leaves and flower buds 10–25%, 2 = visual damage on leaves and flower buds 26%–50%, 3 = visual damage on leaves and flower buds 51–75%, and 4 = visual damage on leaves and flower buds 76–100%.

#### 2.7.4. Natural Enemies Assessment

Ladybird beetles, spiders, lacewing, and parasitoids were the natural enemies of *A. craccivora* encountered in this study. Apart from parasitoid, the other natural enemies were assessed by counting their numbers on randomly selected cowpea plants in each plot. Parasitoids were assessed by collecting 20 mummies per plot through active sampling, and were transferred into perforated petri-dishes and kept at room temperature. The number of parasitoids that emerged from the mummies was recorded, and parasitism rate was computed as per each treatment.

#### 2.7.5. Leaf Vegetable and Grain Yield

Cowpea leaf vegetable yield data were collected weekly, starting from day 21 after planting [42] for 6 weeks. Twenty randomly selected plants in each replicate (80 plants in 4 replicates) were assessed for leaf yield, while all the plants (approximately 500 plants) in each replicate were assessed for grain yield. The total leaf vegetable weight for each treatment was calculated by pooling together the fresh leaf weights obtained for every treatment at the different leaf harvesting dates, expressed in kilograms per hectare (kg·ha^−1^). Dry cowpea grain yield was obtained by picking mature pods, sun drying and threshing, and recording the leaf and grain weight using electronic weighing balance, with yield computed and expressed in kilograms per hectare (kg·ha^−1^).

### 2.8. Statistical Analysis

The aphid infestation density and natural enemies count data, aphid damage assessment score data, and leaf and grain yield data were first log transformed before subjecting the data to analysis using a generalized linear mixed model and means separated using Tukey honestly significant difference (HSD). Aphid mortality data induced by the *M. anisopliae* ICIPE 62 were corrected for natural mortality [43], tested for normality using the Shapiro–Wilk test [44], arcsine transformed in case the data were normalized before subjecting the data to analysis of variance (ANOVA), and means separated using Tukey HSD. Leaf and grain yield data were back-transformed and expressed in kilograms per hectare. Two-sample *t*-test was used to compare *M. anisopliae* ICIPE 62 efficacy and mycosis rates in cowpea monocrop treated with ICIPE 62 and cowpea–maize intercrop treated with the fungus. Data were analyzed using R software version 3.6.1 [45].

## 3. Results

### 3.1. Effect of Cowpea–Maize Intercropping and Treatment Application on Aphid Infestation

**Figure 1:**
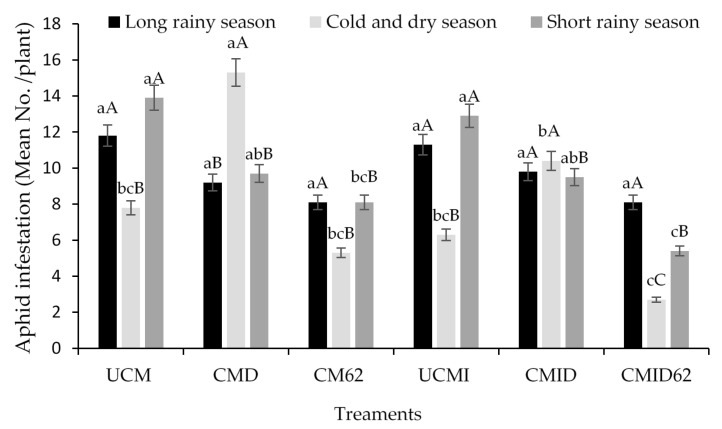
Mean aphid population density (number of aphids per plant) after treatment for the three seasons. UCM (untreated cowpea monocrop), UCMI (untreated cowpea–maize intercrop), CM62 (cowpea monocrop treated with *Metarhizium anisopliae* ICIPE 62), CMI62 (cowpea–maize intercrop treated with *M. anisopliae* ICIPE 62), CMD (cowpea monocrop treated with Duduthrin), and CMID (cowpea–maize intercrop treated with Duduthrin). Data presented are mean ± SE at *P* < 0.05. Means followed by the same lowercase letters and uppercase letters are not significantly different within a season and across/among seasons, respectively.

In the long rainy season, 6 weeks of treatment application did not result in any significant difference (*F* = 1.57, df = 5, *P* = 0.18) in aphid infestation (number of aphids per plant) among the various treatments and the controls, even though untreated cowpea monocrop as well as untreated cowpea–maize intercrop recorded higher infestation levels (Figure 1). However, during the cold and dry season, the aphid infestation varied significantly between the treatments (*F* = 7.2, df = 5, *P* < 0.001), with high infestation levels obtained in both monocrop (15.3 ± 5.8) and the intercrop (10.4 ± 3.4) treated with Duduthrin compared to other treatments. In addition, when comparing the infestation rates across seasons, aphid population densities were higher in long and short rainy seasons than cold and dry season, except for the Duduthrin treatments and the intercrop (Figure 1). A similar trend was observed in the short rainy season where the treatment application resulted in significant reduction (*F* = 8.2, df = 5, *P* < 0.001) in aphid infestation levels, with the lowest infestation recorded in the cowpea–maize intercrop treated with *M. anisopliae* ICIPE 62, whereas the highest was recorded in untreated cowpea monocrop and untreated cowpea-maize intercrop (Figure 1). Comparison of aphid infestation across the three seasons showed that seasons had a significant interaction effect (*F* = 138; df = 2; *P* < 0.001) on aphid infestation with less aphid density (2.7 ± 1) recorded in cold and dry season compared to the other two seasons. Similarly, there was positive interaction between the seasons and the various treatments with regard to aphid population density per plant (*F* = 1.67, df = 10, *P* = 0.008).

### 3.2. Aphid Mortality and Mycosis Induced by Metarhizium Anisopliae ICIPE 62 Application in the Field

Mortality induced by *M. anisopliae* ICIPE 62 was evaluated for cowpea monocrop and cowpea–maize intercrop treated with ICIPE 62 for all the seasons. The mortality induced by ICIPE 62 ranged between 80.4 ± 3.1% and 88.1 ± 2.2% in cowpea monocrop treated with ICIPE 62 and between 79.2 ± 3% and 88.5 ± 2.3% in cowpea–maize intercrop treated with the fungus. Similarly, the mycosis rates followed the same trend, where the rates ranged between 75.2 ± 3.8% and 82.3 ± 2.2% in cowpea monocrop treated with ICIPE 62 and between 72.6 ± 4.0% and 84.4 ± 1.9% in cowpea–maize intercrop treated with the fungus. There were no significant differences between the two fungal treatments with regards to aphid mortality (*t*-values ranged between 0.24 and 0.39, and *P*-values ranged between 0.65 and 0.80) and mycosis (*t*-values ranged between 1.9 and 2.8; and *P*-values ranged between 0.75 and 0.81)) in all the three seasons.

### 3.3. Natural Enemies Associated with Aphis Craccivora

The natural enemies of *A. craccivora* that were observed in the three seasons included ladybird beetles, spiders (*Leucocage decorata*), lacewing, and parasitoid (*Aphidius colemani*). In the long rainy season, the ladybird beetle densities were significantly different among the treatments (*F* = 3.4, df = 5, *P* = 0.04), with the highest numbers recorded in untreated cowpea monocrop and untreated maize–cowpea intercrop. During the cold and dry season, the number of ladybird beetles was significantly different between the treatments (*F* = 13.9, df = 5, *P* < 0.001), with untreated and fungal treatments recording the highest densities. Similarly, in the short rainy season, there was significant difference (*F* = 4.48, df = 3, *P* = 0.001) among the treatments, with the highest number of ladybird beetles obtained in untreated cowpea monocrop (Table 1). There were no significant differences between the treatments in the number of *L. decorata* during the long rainy season (*F* = 1.34, df = 5, *P* = 0.25) and the cold and dry season (*F* = 1.23, df = 5, *P* = 0.3) (Table 1). However, the number of *L. decorata* in the short rainy season was significantly different between the treatments (*F* = 2.45, df = 5, *P* = 0.03), with the lowest number recorded in cowpea monocrop treated with ICIPE 62 (Table 1). The lacewing numbers observed in the long rainy season were significantly different between the treatments (*F* = 1, df = 5, *P* = 0.04), with the highest mean densities obtained in untreated cowpea monocrop. In the dry and cold season, the lacewing numbers observed were significantly different among the six treatments (*F* = 2.6, df = 5, *P* = 0.02), with a larger number obtained in cowpea–maize intercrop treated with ICIPE 62 and Duduthrin. However, the application of the different treatments in the short rainy season did not produce any significant differences (*F* = 1.22, df = 5, *P* = 0.3) in the number of lacewing (Table 1). The aphid parasitoid (*A. colemani*) numbers were the lowest across all the seasons among the collected natural enemies of *A. craccivora*, but were higher in the long rainy season compared to other seasons. However, there were no significant differences among the treatments during the cold and dry (*F* = 0.5, df = 5, *P* = 0.78), long rainy (*F* = 0.11, df = 5, *P* = 0.1), and short rainy (*F* = 1.48, df = 5, *P* = 0.19) seasons (Table 1). This implies the abundance of the parasitoid was not affected by the seasons but rather by other factors.

### 3.4. Aphid Damages on Cowpea

Damage caused by aphids on cowpea plants were not significantly different (*F* = 1.22, df = 5, *P* = 0.34) in the long rainy season. In the cold and dry season, the least damage on cowpea was observed in cowpea monocrop treated with ICIPE62, whereas the highest damage was recorded in cowpea monocrop treated with Duduthrin with highly significant differences (*F* = 6, df = 5, *P* = 0.001) among the treatments (Table 2). The damage caused by aphids differed significantly in the short rainy season (*F* = 4.6, df = 5, *P* < 0.001) among the various treatments, and the cowpea monocrop treated with ICIPE 62 offered better protection against the aphid population because it recorded the least damage (Table 2). Season-wise comparison revealed that the damage differed significantly among the three seasons (*F* = 4.81, df = 2, *P* = 0.008), with a high damage level in untreated plots compared to cowpea maize intercrop treated with ICIPE 62, especially in cold–dry and short rainy seasons. The seasonal variability also significantly influenced (*F* = 4.48, df = 5, *P* < 0.001) the efficacy of the treatments. In addition, there was significant interaction effects (*F* = 2.21; df = 10; *P* = 0.01) between the seasons and treatments.

### 3.5. Leaf and Grain Yield

There was significant difference (*F* = 3.0, df = 5, *P* < 0.001) in the leaf yield among the treatments during the long rainy season. The green leaf yield in the long rainy season was high in the untreated cowpea monocrop and untreated cowpea–maize intercrop compared to other treatments (Table 3). In the cold and dry season, among the monocrop cowpea systems, the cowpea monocrop treated with ICIPE 62 recorded the highest leaf yield (139.56 ± 18 kg·ha^−1^), whereas in the intercrop cluster, the cowpea–maize intercrop treated with ICIPE 62 recorded the highest green leaf yield (106.6 ± 15.7 kg·ha^−1^), with significant difference (*F* = 6.3, df = 5, *P* = 0.001) between the treatments (Table 3). In the short rainy season, the green leaf yields were generally lower compared to the cold and dry season, but higher than the ones obtained in the long rainy season. The green leaf yields differed significantly (*F* = 2.12, df = 5, *P* = 0.01) among the six treatments during the short rainy season (Table 3). The green leaf yield was significantly influenced (*F* = 63.37, df = 2, *P* < 0.001) by prevailing weather conditions, as shown in the comparison among the various seasons. Season also affected the efficacy of the treatments, and there was significant difference (*F* = 4.74, df = 2, *P* < 0.001) in the treatments across the seasons (Table 3). In addition, there was also a positive interaction between the season and treatment (*F* = 2, df = 10, *P* = 0.008).

In the long rainy season, the cowpea grain yield was significantly different (*F* = 4.26, df = 5, *P* < 0.001) among the six treatments (Table 3). The highest grain yield was obtained in cowpea monocrop treated with ICIPE 62 (302 ± 31.8 kg·ha^−1^), whereas the lowest was recorded in untreated cowpea–maize intercrop (183.7 ±16.9 kg·ha^−1^). However, the treatment application did not influence much of the grain yield in the monocrops, and the same trend was observed in the intercrop systems (Table 3). In the cold and dry season, the cowpea monocrop treated with ICIPE 62 recorded the highest grain yield (256.8 ± 23.4 kg·ha^−1^) compared to other treatments, with highly significant difference (*F* = 16.15, df = 5, *P* < 0.001) among the various treatments. In the short rainy season, the grain yields were generally lower compared to the long rainy and cold and dry seasons. However, there was significant difference (*F* = 5.67, df = 5, *P* = 0.001) among the treatments with regard to the grain yield, with the highest yields recorded in cowpea monocrop treated with ICIPE 62 (156.5 ± 17.5 kg·ha^−1^) and cowpea–maize intercrop treated with ICIPE 62 (125.3 ± 17.4 kg·ha^−1^) (Table 3). Furthermore, the grain yield was significantly influenced (*F* = 86, df = 2, *P* < 0.001) by the season type. The season also influenced the performance of the different treatments (*F* = 16.97; df = 2; *P* < 0.001), and there was a positive interaction (*F* = 2.58, df = 10, *P* = 0.005) between the prevailing environmental conditions and the treatment efficacy (Table 3). Comparison of leaf yield means between fungus-treated plots in all the three seasons showed that the monocrop yielded more (123.5 ± 12.4 kg·ha^−1^) than intercrop (88.9 ± 9.1 kg·ha^−1^), and there was significant difference (*F* = 13.43, df = 1, *P* < 0.001) between the treatments. The gain yield for monocrop was (238.6 ± 16.8 kg·ha^−1^) and intercrop (94.63 ± 13.6 kg·ha^−1^), and the yields differed significantly (*F* = 11.1, df = 1, *P* < 0.001).

## 4. Discussion

In this study, the long rainy season recorded a late infestation of aphids. During this season, all the treatments did not reduce aphid infestation, although untreated cowpea monocrop and untreated cowpea–maize intercrop recorded slightly higher aphid infestations. In the cold and dry season, there was reduced rainfall, leading to early and heavy aphid infestation, where the treatment effects on the aphid population was noticeable. The combination of cowpea–maize intercrop treated with *M. anisopliae* ICIPE 62 was more effective in reducing aphid infestations per plant by recording the least aphid density after 6 weeks of treatment. Our results also showed that intercropping cowpea and maize without application of ICIPE 62 or Duduthrin (a synthetic pyrethroid) did not reduce aphid infestations in all three seasons. Duduthrin, which is registered in Kenya for aphid management, did not effectively control the aphids either in the monocrop or in the intercrop. Previous studies have reported similar findings, wherein Duduthrin application did not result in reducing *A. craccivora* infestations or effectively controlling them [17]. This could be due to previously possible unreported development of resistance by *A. craccivora* against the pesticide. Aphids are known to develop resistance to chemicals after years of exposure [15,46]. Therefore, research on improvement of this strategy (continuous use of chemical pesticides) has been developed and has focused on a combination of several strategies such as intercropping and monitored application of pesticides [22,23]. However, deleterious effects of chemical pesticides have encouraged a search for safer alternatives of pest management. Furthermore, the low efficacy of Duduthrin against *A. cracivora* could not be adequately explained in the current study; however, we recommend further research to understand factors that drove this with emphasis placed upon possible resistance development by the insect against the insecticide after several years of applications in Kenya.

The good performance of the combination of cowpea–maize intercrop and application of *M. anisopliae* ICIPE 62 could be attributed to many factors. Intercropping results in increased relative humidity and reduced light penetration into the lower canopy crop [47,48]. Performance of EPF-based biopesticides is known to be affected by relative humidity and UV light [49,50,51]. Therefore, improved relative humidity and reduced fungal spore degradation due to light interception in the intercrop could have enhanced the efficacy of the EPF, resulting in better performance. Similar findings were reported by Ekesi et al. [52], who demonstrated better control of legume flower thrips *Megalurothrips sjostedti* Trybom (Thysanoptera: Thripidae) in cowpea intercropped with maize and treated with EPF (*M. anisopliae*).

In the short rainy season, which recorded a slightly higher rainfall than the cold and dry season but lower than the long rainy season, a similar trend was observed, wherein lower aphid infestations were recorded after 6 weeks of treatment application. Even though the intercropping strategy in insect pest management including aphids has been widely researched [4,53,54], studies including the present one have demonstrated that use of this strategy alone does not guarantee successful pest management. Application of ICIPE 62-based biopesticide in cowpea–maize intercrop systems could provide alternatives to use of synthetic chemical pesticides, and consequently could provide a more sustainable management of aphids because the biopesticides are user-friendly, safe for the environment, and have lower negative impacts on natural enemies of aphids compared to synthetic insecticides. The *M. anisopliae* isolate ICIPE 62 used in this study has been previously known to be pathogenic to several aphid species, both in the laboratory and in the field, including *A. craccivora* [17,30,55], and has been developed by *icipe* and commercialized by Real IPM for use in management of aphids (http://www.realipm.com/). Therefore, the results of this study gave more evidence to extend the label of this *M. ansipliae* ICIPE 62-based biopesticide to the management of *A. craccivora.* Although EPF-based biopesticides are slow-acting and do not produce immediate control or quick knockdown and their efficacy is dependent on environmental conditions [50,56,57], they can be used in integrated management systems where intercropping could provide high protection of the conidia.

During the long rainy season, the treatments had no effect on the damage of cowpea by *A. craccivora*, as there was no difference between the treatments. However, untreated cowpea monocrop recorded higher damage compared to other treatments. In the cold and dry season where aphid infestation occurred early after crop emergence, the damage on the cowpea crop was higher in cowpea monocrop treated with Duduthrin, whereas cowpea–maize intercrop treated with ICIPE 62 recorded the least damage. In the short rainy season, all the treatments recorded similar damage, except the cowpea–maize intercrop treated with ICIPE 62, which provided adequate protection against *A. craccivora*. Intercropping cowpea and cereals such as maize and sorghum has many advantages, including increased yield, improvement in soil fertility, better utilization of resources, as well as insect pest management [58,59]. In intercropped systems, pests are visually disturbed and tend to stay for shorter times on the hosts due to the disruptive effect of landing on non-host plants, which reduces their survival and damage [60,61]. It was also reported that intercropping cowpea and cereals creates a physical barrier against aphids [62]. In this study, intercropping alone without application of ICIPE 62 or intercropping and application of Duduthrin did not protect the crop against damage by aphids. The cowpea landrace used in this study is known to have high susceptibility to *A. craccivora*, and in susceptible germplasms, damage is bound to occur even at low levels of infestation [62]. Aphid population builds up fast within a short period of time in favorable environmental conditions due to their reproductive nature (parthenogenesis and viviparity), and this increases damage incidences in susceptible plants [63]. The inability of the intercrop to reduce aphid infestation could also be attributed to the cropping practice used in this study. Cowpea and maize were planted simultaneously, and hence the maize could not offer the pest barrier or the visual disturbance effects [53,62]. Even though it has been shown that staggered planting is able to protect cowpea from insect pests in an intercrop system [22,53], the applicability of this cropping system is not feasible in arid and drier areas where cowpea is a major crop and early and simultaneous cropping of multiple crops is the norm. This practice enables the crops to benefit from the little available moisture and assures farmers of some harvest especially when the rainfall is inadequate.

During the three seasons, the natural enemies of *A. craccivora* we encountered were ladybird beetles, spiders (mainly *Leucauge decorata*), lacewing, and parasitoid (*Aphidius colemani*), and their numbers/densities varied across the treatments. In the long rainy season, the highest number of ladybird beetles was recorded in untreated cowpea monocrop, and the least was obtained in cowpea monocrop treated with Duduthrin. Cold and dry season recorded the highest number of ladybirds among all the three seasons. This could be attributed to the fact that aphid infestation occurred early in the season and the population buildup was higher, meaning the aphid population could sustain a higher number of the predatory ladybird beetles, whereas in the short rainy season, the highest number of ladybird beetles was obtained in untreated cowpea monocrop plots. Across the three seasons, spider population was higher in the long rainy season compared to the cold–dry and short rainy seasons. Although the untreated cowpea monocrop recorded a lower population of *L. decorata* in the long rainy season, the observation was different in the other two seasons.

The lacewing population was highest in the long rainy season but lower in the cold–dry and short rainy seasons. In the wet season, the untreated cowpea monocrop recorded a higher population of lacewing among the treatments. The variation in the population of lacewings could be related to the seasons, as the aphid infestations during the long rainy seasons were not significant among the treatments, but a high number of the predator was obtained.

The parasitoid *A. colemani* was the lowest in terms of population density among the natural enemies and did not appear to be affected by the treatments. The lower number of the parasitoid could be attributed to the sensitivity of the genus to pesticides [64], as the experimental site has been previously used for different experiments for a long time, where some plots were involved in the use of synthetic pesticides; this could have reduced their population gradually as per the practice history of the experimental plots.

In intercropping systems, cowpea cushions the farmers in case of cereals crop failure because they can harvest the cowpea [65]. In the present study, both cowpea leaf and grain yield were evaluated for the three seasons. In the long rainy season, the leaf yield was higher in monocrop systems compared to intercrops, irrespective of the applied treatments, except in ICIPE 62 treatments. The higher leaf yield in monocrops was attributed to higher cowpea plant density in monocrops compared to intercrops.

In the cold and dry season, aphid infestations occurred early after crop emergence (7 days after emergence (DAE)), and treatment applications commenced 14 after planting. Cowpea monocrop treated with ICIPE 62 recorded higher leaf yield among the monocrops and overall. This implies that the treatment could protect the crop after early and heavy infestation by *A. craccivora* and produce higher yields. Duduthrin did not confer yield advantage as expected. This could partly be explained by the fact that the treatment recorded highest aphid density (aphids per plant) and consequently the highest plant damage. Aphid damage on leaf yield is not only through direct feeding on the leaves but also affects quality due to production of honeydew on the leaves that also reduces marketable leaf yield. In the short rainy season, aphid infestation occurred 21 DAE, and treatment commenced immediately thereafter. The season was characterized by intermittent rainfall, and the leaf yield significantly differed between the treatments, but ICIPE 62 treatments in both monocrops and intercrops recorded higher yields. The application of Duduthrin was not effective in reducing either aphid population, and hence did not confer the leaf yield advantage.

In the short rainy season, cowpea–maize intercrop treated with ICIPE 62 produced higher yield, whereas maize intercrop treated with ICIPE 62 and Duduthrin produced similar yields. *Aphis craccivora* attacks cowpea in all stages of its development, from seedling to podding stage, and is one of the key limiting factors in cowpea production [10,41]. Therefore, timing of application of control measures is critical in reversing yield losses. Although performance of EPF-based biopesticides is limited by their slow-acting nature and environmental factors [50,57,58,66], the current study demonstrated that with proper timing of control interventions coupled with favorable weather conditions, the EPF-based biopesticides can offer effective control and confer yield benefits to farmers. The *M. anisopliae* ICIPE 62 has been demonstrated to be pathogenic to several aphid species under laboratory, screenhouse, and field conditions [17,30,67], and consequently contributed to yield gain.

This study therefore demonstrated the potential of this *M. anisopliae* isolate to control *A. craccivora* under field conditions in an integrated management approach. The isolate also did not negatively affect the associated natural enemies of *A. craccivora*, and this presents another advantage for the commercialization of *M. anisopliae* ICIPE 62. The non-target effect observed in this study has been reported earlier under laboratory conditions [67]. Cowpea is usually grown for dual-purposes, that is, leafy vegetables and grain [6], and this presents an advantage because farmers can derive dual benefits from the yields. Where cowpea is grown as a vegetable, the practice is to uproot the crop after 4–6 weeks, and this means the grain yield is lost; hence, this study recommends the practice of picking leaves rather than uprooting. Harvesting leaves at specific intervals has been shown to positively influence the grain yield [65], but confers farmers with more benefits.

## 5. Conclusions

In conclusion, this study demonstrated the efficacy of combination of intercropping and application of EPF as an alternative approach that can be used in the management of aphids on vegetables. *M. anisopliae* ICIPE 62 did not negatively affect the natural enemies of *A. craccivora*, and this represents an added advantage for its commercialization. New and improved application strategies such as good timing of application of control measures is critical to improve cowpea yield. Use of synthetic pesticides in vegetables such leafy cowpea is disadvantageous because of the need to observe post-harvest intervals (PHI). Vegetables are harvested frequently, and use of the synthetic pesticides leads to yield loss during observation of PHIs. During off-season production when the leafy vegetable is in high demand, application of pesticides increases food safety risks because farmers and consumers may not adhere to PHI guidelines besides the environmental pollution and killing of non-target beneficial arthropods by the pesticides.

## Figures and Tables

**Table 1:** Beneficial arthropod density recorded in the various treatments during the three seasons.

Seasons		Means ± SE			
	Treatment	Lady Bird Beetles	Spiders (*Leucauge decorata)*	Lacewing	Parasitoid (*Aphidius colemani*)
Long rainy season	UCM	2.7 ± 0.4 a	1.6 ± 0.3 a	2.6 ± 0.6 a	0.5 ± 0.2 a
	CMD	1.3 ± 0.3 b	2.3 ± 0.8 a	1.8 ± 0.5 b	0.3 ± 0.2 a
	CM62	1.5 ± 0.3 ab	2.4 ± 0.5 a	1.7 ± 0.3 b	0.5 ± 0.2 a
	UCMI	2.3 ± 1.0 ab	2.7 ± 0.5 a	1.2 ± 0.3 b	0.4 ± 0.2 a
	CMID	1.3 ± 0.5 b	1.3 ± 0.3 a	1.4 ± 0.4 b	0.2 ± 0.1 a
	CMI62	1.6 ± 0.3 ab	2.8 ± 0.6 a	1.7 ± 0.4 b	0.5 ± 0.1 a
Cold and dry season	UCM	10.6 ± 2.2 a	1.9 ± 0.5 a	0.4 ± 0.2 ab	0.2 ± 0.1 a
	CMD	10.1 ± 1.7 a	1.2 ± 0.4 a	0.5 ± 0.2 ab	0.2 ± 0.1 a
	CM62	6.5 ± 1.1 a	0.8 ± 0.3 a	0.3 ± 0.2 b	0.2 ± 0.1 a
	UCMI	2.6 ± 0.8 b	1.4 ± 0.3 a	0.5 ± 0.2 ab	0.1 ± 0.0 a
	CMID	10.4 ± 1.5 a	2.2 ± 0.5 a	1.0 ± 0.4 a	0.2 ± 0.1 a
	CMI62	6.5 ± 1.1 a	1.2 ± 0.2 a	1.2 ± 0.2 a	0.1 ± 0.1 a
Short rainy season	UCM	3.9 ± 0.7 a	1 ± 0.2 a	0.6 ± 0.2 a	0.02 ± 0.01 a
	CMD	2.9 ± 0.1 ab	0.7 ± 0.2 ab	0.3 ± 0.1 a	0.07 ± 0.01 a
	CM62	2.0 ± 0.5 b	0.3 ± 0.1 b	0.2 ± 0.1 a	0.32 ± 0.02 a
	UCMI	2.0 ± 0.5 b	0.5 ± 0.1 ab	0.1 ± 0.06 a	0.00 ± 0.0 a
	CMID	2.5 ± 0.6 b	0.7 ± 0.2 ab	0.3 ± 0.2 a	0.09 ± 0.01 a
	CMI62	2.4 ± 0.5 b	0.4 ± 0.1 ab	0.2 ± 0.1 a	0.14 ± 0.05 a

Means followed by the same letter within a column are not significantly different by Tukey’s honestly significant difference (HSD) at *P* < 0.05. UCM (untreated cowpea monocrop), UCMI (untreated cowpea–maize intercrop), CM62 (cowpea monocrop treated with *M. anisopliae* ICIPE 62), CMI62 (cowpea–maize intercrop treated with *M. anisopliae* ICIPE 62), CMD (cowpea monocrop treated with Duduthrin), and CMID (cowpea–maize intercrop treated with Duduthrin).

**Table 2:** Cowpea plant damages as influenced by the different treatments during the three seasons using the ranking scale.

Treatment	Long Rainy Season	Cold and Dry Season	Short Rainy Season
UCM	1.9 ± 0.2 aA	1.2 ± 0.2 abB	2.0 ± 0.2 aA
CMD	1.6 ± 0.2 aB	2.1 ± 0.3 aA	1.7 ± 0.3 aA
CM62	1.3 ± 0.2 aA	1.0 ± 0.2 bA	1.2 ± 0.2 abA
UCMI	1.6 ± 0.2 aA	1.0 ± 0.2 bB	2.0 ± 0.3 aA
CMD	1.5 ± 0.2 aA	1.1 ± 0.3 bA	1.7 ± 0.3 aA
CMID	1.6 ± 0.3 aA	0.4 ± 0.2 cC	0.8 ± 0.1 bB
*F*	1.22	6	4.6
*P*-Value	0.34	< 0.001	< 0.001
df	5	5	5

Means followed by the same lowercase letters within a column and same uppercase letters within a row were not significantly different by Tukey HSD at *P* < 0.05. UCM (untreated cowpea monocrop), UCMI (untreated cowpea–maize intercrop), CM62 (cowpea monocrop treated with *M. anisopliae* ICIPE 62), CMI62 (cowpea–maize intercrop treated with *M. anisopliae* ICIPE 62), CMD (cowpea monocrop treated with Duduthrin), and CMID (cowpea–maize intercrop treated with Duduthrin).

**Table 3:** Cowpea leaf and grain yield (kg/ha) as influenced by the different treatments during the three seasons.

Yield	Treatment	Long Rainy Season	Cold and Dry Season	Short Rainy Season
Leaf yield (kg·ha^−1^)	UCM	16.8 ± 2.4 aB	69.6 ± 5.5 bA	22.65 ± 5.4 bB
	CMD	14.6 ± 1.7 abB	62.38 ± 5.3 bA	26.65 ± 13.5 bB
	CM62	9.3 ± 1.3 bC	139.56 ± 18.7 aA	46.4 ± 11.6 aB
	UCMI	16.8 ± 2.4 aB	89.44 ± 9.3 bA	25.4 ± 6.6 bB
	CMID	11.8 ± 1.7 bC	80.4 ± 6.6 bA	31.55 ± 8.3 abB
	CMI62	10.2 ± 1.7 bC	106.6 ± 15.7 abA	40.45 ± 10.7 aB
	*F*	3	6.25	2.12
	*P*-Value	< 0.001	< 0.001	0.01
	df	5	5	5
Grain yield (kg·ha^−1^)	UCM	285.7 ± 3 abA	139.6 ± 15 bcB	102.9 ± 11 bC
	CMD	277.9 ± 32 abA	117.33 ± 7 bcB	97.82 ± 4.8 bB
	CM62	302 ± 31.8 aA	256.8 ± 23.4 aA	156.5 ± 17.5 aB
	UCMI	183.7 ± 16.9 bA	84.9 ± 7 cB	74.9 ± 7.2 bB
	CMID	200 ± 27 abA	83.2 ± 8.4 cB	87.39 ± 10 bB
	CMI62	203.17 ± 32 abA	150.7 ± 23.5 bB	125.3 ± 17.4 abB
	*F*	4.26	16.15	5.67
	*P*-Value	< 0.001	< 0.001	< 0.001
	df	5	5	5

Means followed by the same lowercase letter within a column and same uppercase letter within a row were not significantly different by Tukey HSD at *P* < 0.05. UCM (untreated cowpea monocrop), UCMI (untreated cowpea–maize intercrop), CM62 (cowpea monocrop treated with *M. anisopliae* ICIPE 62), CMI62 (cowpea–maize intercrop treated with *M. anisopliae* ICIPE 62), CMD (cowpea monocrop treated with Duduthrin), and CMID (cowpea–maize intercrop treated with Duduthrin).
